# Imported Monkeypox from International Traveler, Maryland, USA, 2021

**DOI:** 10.3201/eid2808.220726

**Published:** 2022-08

**Authors:** Faisal S. Minhaj, Agam K. Rao, Andrea M. McCollum

**Affiliations:** Centers for Disease Control and Prevention, Atlanta, Georgia, USA

**Keywords:** Monkeypox, poxvirus, Orthopoxvirus, viruses, public health, United States

**To** **the Editor:** Costello et al. described a patient in Maryland, USA, with a diffuse vesicular rash initially diagnosed as disseminated varicella zoster virus (VZV) infection. Only after a biopsy revealed unexpected findings was monkeypox suspected (*1*). Monkeypox is commonly confused with VZV in countries where both infections are endemic. High fever, lymphadenopathy, and a deep-seated, well-circumscribed, umbilicated rash in the same stage of development (i.e., macule, papule, vesicle, or scab) in distinct anatomic locations are characteristic of monkeypox (*2*). Although the patient in Maryland experienced lymphadenopathy and rash with umbilicated lesions suggestive of monkeypox, he was afebrile, denied other prodromal signs and symptoms (e.g., headache and chills) that typically precede monkeypox rash, and improved while receiving intravenous acyclovir, features more consistent with VZV. However, the unusual clinical signs and symptoms experienced by this patient were similar to those observed in other patients in the evolving 2022 multinational monkeypox response. 

Because differential diagnosis can be challenging, public health authorities should be consulted promptly when monkeypox is possible. US Laboratory Response Network laboratories (https://emergency.cdc.gov/lrn) can enable rapid testing of specimens (e.g., lesions swab), and pathogen-specific antiviral medications can be acquired through consultation with the Centers for Disease Control and Prevention. Public health investigation for a single case of monkeypox can be intensive and complicated; case-patient contacts outside of the hospital must be identified, monitored, and potentially given 1 of the 2 orthopoxvirus vaccines offered for postexposure prophylaxis in the United States (*3**–**5*).

Factors that should raise suspicion for monkeypox in a patient with related signs and symptoms include history of travel outside of the United States to a country with confirmed cases or where monkeypox virus is endemic, contact with a person with a similar-appearing rash or who has received a diagnosis of confirmed or probable monkeypox, contact with Africa-endemic wild animal or pet species (living or dead), or use of a product derived from those animals (e.g., game meat, creams, lotions, powders). Monkeypox should also be considered in patients with close or intimate contact with persons in social networks experiencing high monkeypox activity, including men who have sex with men who meet partners through a website, digital application, or social event. Prompt consultation with public health authorities is essential for providing clinical guidance, expediting testing and treatment, and preventing secondary cases (*3*).
